# RelB is a potential molecular biomarker for immunotherapy in human pan-cancer

**DOI:** 10.3389/fmolb.2023.1178446

**Published:** 2023-06-14

**Authors:** Jintao Wu, Xinyu Yu, Hongyu Zhu, Peng Chen, Tongyan Liu, Rong Yin, Yan Qiang, Lin Xu

**Affiliations:** ^1^ Jiangsu Key Laboratory of Molecular and Translational Cancer Research, Department of Thoracic Surgery, Jiangsu Cancer Hospital & Nanjing Medical University Affiliated Cancer Hospital & Jiangsu Institute of Cancer Research, Nanjing, China; ^2^ Department of Science and Technology, Jiangsu Cancer Hospital & Nanjing Medical University Affiliated Cancer Hospital & Jiangsu Institute of Cancer Research, Nanjing, China; ^3^ Biobank of Lung Cancer, Jiangsu Biobank of Clinical Resources, Nanjing, China; ^4^ Collaborative Innovation Center for Cancer Personalized Medicine, Nanjing Medical University, Nanjing, China; ^5^ Department of Intensive Care Unit, Nanjing Medical University Affiliated Cancer Hospital & Jiangsu Institute of Cancer Research, Nanjing, China

**Keywords:** pan-cancer, immune infiltration, prognostic marker, immune checkpoints, RelB/NFκB

## Abstract

**Introduction:** The nuclear factor kB (NF-κB) pathway emerges as a critical regulator of immune responses and is often dysregulated in human cancers. It consists of a family of transcription factors involved in many biological responses. Activated NF-κB subunits results in the nuclear translocation and activation of transcription, and the NF-κB pathway is known to influence the transcription of many genes. Noncanonical NF-κB and its components have been shown to have effects, usually protumorigenic, in many different cancer types. Besides, NF-κB signaling had diverse and complicated roles in cancer with studies that NF-κB could both contribute to tumor promotion and suppression of oncogenesis relying on the cellular context. RelB, a member of noncanonical NF-κB was abnormally regulated in most cancer types, however the molecular features and clinical signature of RelB expression, as well as its role in cancer immunity in human pan-cancer remains to be elucidated.

**Methods:** We used the open databases to explore RelB expression, clinical features and the association with tumor-infiltration cells in human pan-cancer. In this study, we investigated the aberration expression and prognostic significance of RelB, and the correlation with clinicopathological characters and immune cells infiltration in various cancers. The Cancer Genome Atlas (TCGA) and Genotype-Tissue Expression (GTEx) databases were used to analyze the mRNA expression level in different cancer types. Kaplan-Meier analysis and Cox regression were used to explore the prognostic significance of RelB in human pan-cancer. Then we took advantage of the TCGA database to analyze the relationship between RelB expression and DNA methylation, the infiltration of immune cells, immune checkpoint genes, tumor mutation burden (TMB), microsatellite instability (MSI), mismatch repair (MSS).

**Results:** Higher expression of RelB was significantly detected in human cancer tissues and a high level of RelB expression was significantly linked with a worse outcome in LGG, KIPAN, ACC, UVM, LUAD,THYM, GBM, LIHC and TGCT but associated with a favorable overall survival (OS) in SARC, SKCM and BRCA. According to the Human Protein Altas database, RelB was considered as an independent factor in breast cancer and renal cancer prognosis. GSEA results revealed that RelB was involved in many oncogenesisrelated processes and immunity-related pathways. RelB was significantly correlated with DNA methylation in 13 types of cancer. Meanwhile, RelB expression was associated with TMB in 5 types of cancer and MSI in 8 types of cancer. In the final, we analyzed the relationship between RelB expression and immune-infiltration cells in human pan-cancer, which suggested RelB could be a promising therapeutic target for cancer immunotherapy.

**Discussion:** Our study further provided insights into a deeper understanding of RelB as a prognostic biomarker.

## 1 Introduction

Cancer still ranks as a prominent cause of death and a serious problem greatly affecting the public life expectancy worldwide (Sung et al., 2021). Cancer exerts immense physical and financial suffering to the individual patients. Overall, the burden of cancer incidence and mortality is still increasing rapidly in every country. The available cancer treatments include radiotherapy, chemotherapy, targeted therapy and immunotherapy. Although great efforts had been made to improve the clinical outcome and the quality of life, the five-years survival was still poor. Pan-cancer analysis was to analyze the relevant genes expressed in a wide variety of cancers to compare the differences and similarities of the selected gene. It was evident that early detection and local disease manipulation could remarkably improve the 5-year survival in many solid tumors (Klein, 2020). Therefore, it is urgent to identify new prognostic biomarkers and novel cancer treatment strategies. Cancer cells underwent oncogene-induced replication stresses that were different from those underwent by the corresponding normal cells (Jeggo et al., 2016). These stresses caused chronic inflammation, oncogene activation, inhibition of tumor suppressors and at least one genetic mutation, which finally resulted in tumor genesis. Since NF-κB played a critical role in inflammatory and immune responses (Ben-Neriah and Karin, 2011), NF-κB was considered to influence tumor immunity. NF-κB activation was mediated by two different pathways, including the canonical pathway and the noncanonical pathway. The canonical pathway responded quickly to the stresses, while the noncanonical pathway responded slowly but persistently activated to deal with the stimulants. NF-κB family consisted of five members, including NF-κB 1 (p50), NF-κB (p52), RelA(p65), RelB and c-Rel. RelA had been reported to modulate PD-L1 expression in lung cancer cells (Asgarova et al., 2018). Additionally, RelB had been demonstrated to be involved in cancer development and progression, and RelB might determine the response of cancer treatments (Karin, 2006). What is important, RelB and its signaling pathways have become an attracting target for chemotherapeutic approaches. In summary, RelB could be a promising target for antitumor therapy. However, it is still in existence that the precise function and molecular mechanism remain unknown and need to be further explored. Although increasing evidence suggested that RelB played an important role in the oncogenesis of most cancer types (Tegowski and Baldwin, 2018), a comprehensively systematic pan-cancer analysis of RelB had not yet been performed. Therefore, the goal of this study was to explore the expression profile, prognostic value, methylation level of RelB, and potential relationship between RelB expression and immune-related functions in human pan-cancer.

## 2 Materials and methods

### 2.1 The Expression of RelB in Pan-Cancer

The TCGA database, a widely used tool to find out the causes of human cancers and verify some conclusions from bioinformation analyses, was exploited to investigate the expression level of RelB across different cancer types. RNA sequencing data and the corresponding clinical information for patients in TCGA database were downloaded and processed for further analyses. GTEx was a good supplement for the limiting normal tissues in patients from TCGA database. The pictures of the analyses results were drawn with the R software packages ggplot2. 0.

### 2.2 RelB expression and its clinical correlation in human pan-cancer

The UALCAN database (http://ualcan.path.uab.edu) was used to investigate and visualize the relationship between RelB expression and tumor clinical stages. Besides, the DNA methylation level was also checked in matched normal and tumor tissues in human pan-cancer. We also estimated the protein level of RelB in some cancer types in UALCAN database.

### 2.3 Gene set enrichment analysis (GSEA)

The downloaded TCGA database was divided into two groups (RelB^high^ and RelB^low^ group) according to the median value of RelB expression in each cancer type. The differential genes between these two groups were selected. GSEA was performed by R package clusterProfiler with selected gene sets. The comparison of the GSEA is the RelB high-expression and low-expression groups. GSEA was conducted to analyze the biological and molecular functions of RelB in various cancer types. CAMOIP (www:camoip.net) was a good tool for analyzing the expression data and mutation data from TCGA and the ICI-treated projects (Lin et al., 2022). We processed the corresponding data and used the CAMOIP to complete the GSEA plots. The above analyses were conducted using the R package Cluster Profiler with selected RelB gene. The comparison of the GSEA was the high-expression and low-expression of RelB according to the median value of RelB expression. For ridgeplot, it was supported by R package enrichplot (ridgeplot function).

### 2.4 cBioPortal database

The cBioPortal Database (http://www.cbioportal.org/) was a powerful tool widely used to get the human gene mutation profile in specific cancer types. We thus exploited it to obtain the genetic alterations profile of RelB across all 32 cancer types in 10,967 samples of 10,953 patients. The alteration type of RelB included inframe mutation, missense mutation, splice mutation, truncating mutation, structural variant, amplification and deep deletion.

### 2.5 The role of RelB in predicting the prognostic potential in pan-cancer

The Kaplan-Meier method was used to investigate the prognostic potential of RelB in pan-cancer using R package survival and survminer. The survival data was downloaded from TCGA database and then was used to analyze the overall survival (OS), disease-specific survival (DFS), disease-free interval (DFI) and progression-free interval (PFI). Univariate Cox regression analysis was used to analyze RelB-related survival with the R package limma, survival, and forestplot to show the *p* value, HR, and 95% CI.

### 2.6 Relationships Between RelB expression and tumor cell infiltration and immune modulator genes in pan-cancer

We obtained the public data for 33 types of human cancer in TCGA from the UCSC Xena website (http://xena.ucsc.edu/). For reliable immune score evaluation, Timer2.0 database was used to integrate the two common algorithms, including TIMER, and xCell. Heatmaps of the immune infiltration scores or immune modulator genes and RelB expression in different cancer types were generated with Pearson correlation analysis. The horizontal axis in the heatmaps shows the relevant genes, and the vertical axis shows the type of cancer, and the color shows the correlation coefficients. In addition, R software v4.0.2 was used for statistical analysis.

### 2.7 Relationship between RelB expression and TMB/MSI in human cancer

The data for all cancer types was downloaded in TCGA from the GDC data portal website. The value of RelB expression was log2 (x+0.001) transformed and we calculated the corresponding TMB or MSI score of every tumor tissue. Their correlation was then performed using Pearson method. R software v4.0.2 was used for statistical analysis. A *p* value less than 0.05 is considered statistically significant.

## 3 Results

1. RelB Was Significantly Upregulated in Human Pan-Cancer

**FIGURE 1:**
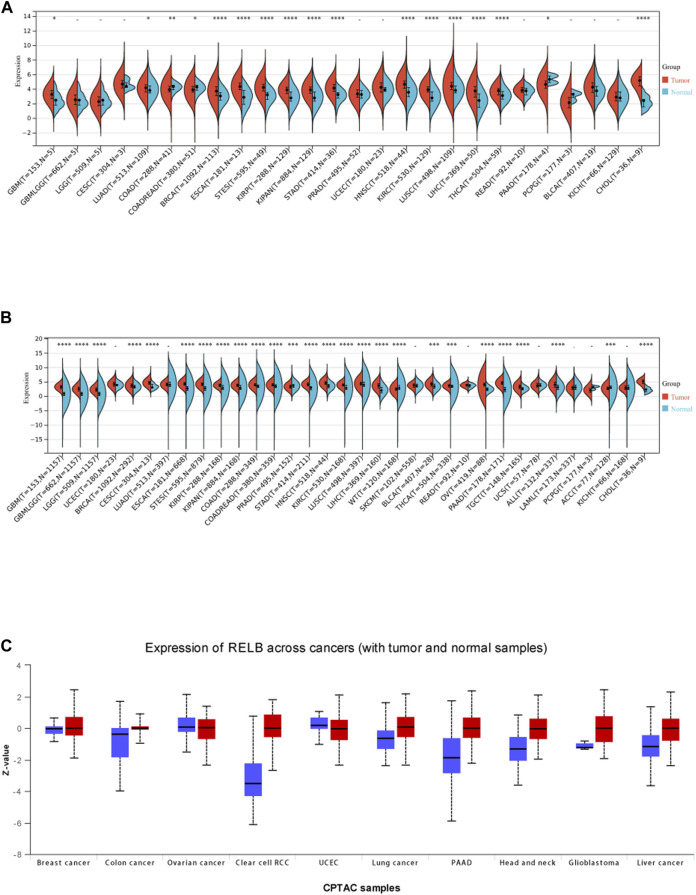
The expression level of RelB in human pan-cancer. **(A)**The expression level of RelB in normal and tumor tissues in human pan-cancer; **(B)** The differential expression of RelB in human pan-cancer in the TCGA and GTEx databases; **(C)**The different protein expression level in matched tumor tissues in the UALCAN database.

According to the analysis results of cancer data from The Cancer Genome Altas (TCGA), we observed RelB expression was significantly upregulated compared with the adjacent normal tissues in GBM, LUAD, COAD, BRCA, ESCA, STES, KIRP, KIPAN, KIRC, LIHC, STAD, HNSC, LUSC, THCA, PAAD, PRAD and CHOL (Figure 1A). Because the normal tissue controls were very limited in some cancer types, we then combined TCGA data and GTEx data together to investigate the RelB expression between cancer tissues and the corresponding normal tissues. We found RelB was highly expressed in cancer tissues in GBM, LGG, BRCA, CESC, ESCA, STES, KIRP, KIPAN, COAD, PRAD, STAD, HNSC, KIRC, LUSC, LIHC, BLCA, THCA, OV, PAAD, TGCT, ALL, ACC and CHOL (Figure 1B). We then evaluated the protein expression of RelB using UALCAN website (Figure 1C). It was evident that the level of RelB protein was higher in LIHC, GBM, HNSC, PAAD, clear cell renal carcinoma and lung cancer, which was consistent with the corresponding mRNA expression in these cancer types. These results implied that RelB might be a pro-tumor role in pan-cancer.

2. Relationship Between RelB Expression and Individual Clinic-Pathological Characteristics Across All Cancer Types

**FIGURE 2:**
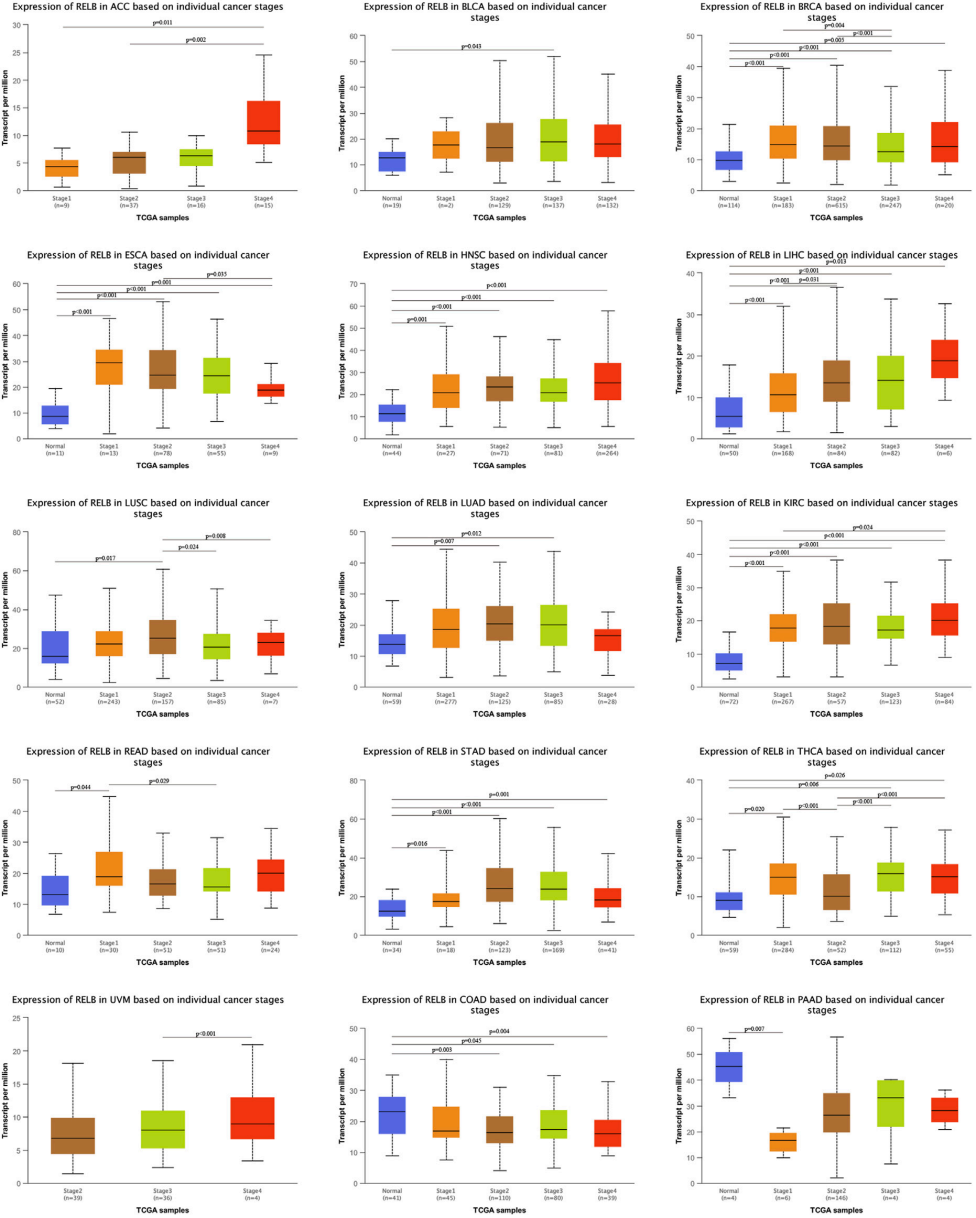
Correlation of RelB expression and clinical cancer stages in different cancer types. The correlation was evaluated using the UALCAN database. RelB was differential expressed in different stages and cancer types. *p < 0.05; **p < 0.01; ***p < 0.001.

The relationship between RelB expression and clinic-pathological features in TCGA database was evaluated based on individual cancer stages. The tumors were classified into stages I, II, III, and IV according to the its clinical information. Compared with normal tissue controls, RelB expression was higher in BRCA, KIRC, ESCA, LIHC, ACC, STAD (Figure 2). However, RelB expression was clearly lower in COAD, PAAD (Figure 2). We also found RelB was stably expressed in HNSC, LIHC and KIRC. In one word, RelB was differently expressed and significantly correlated with tumor stages in most cancer types.

3. Prognostic Value of RelB in Human Pan-Cancer

**FIGURE 3:**
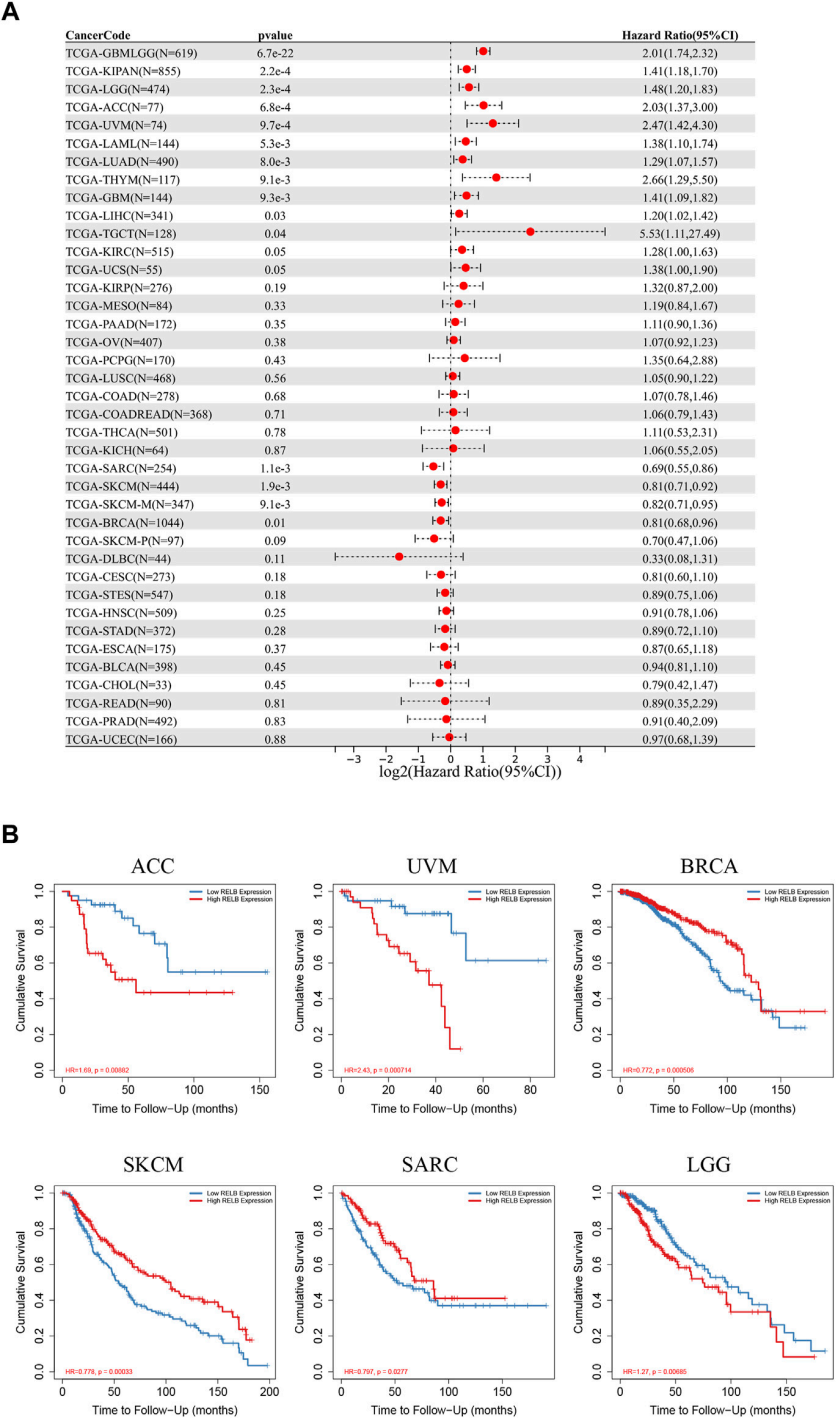
The prognostic role of RelB in human pan-cancer. **(A)** Relationship between RelB expression and overall survival (OS) using the Cox regression method in pan-cancer. **(B)** OS curves comparing high and low expression of RelB in some cancer types with Kaplan-Meier methodology in TIMER2.0 database.

The pan-cancer patients were divided into two groups according to the median expression of RelB expression in various cancer types. We next estimated the relationship between RelB expression level and the prognosis of the patients in TCGA database, including overall survival (OS), disease-specific interval (DFI) and progression-free interval (PFI). Based on the OS cox-regression analysis results in the forest plot (Figure 3A), high RelB-expression was closely related to a poorer OS in most cancer types, including LGG, GBM, KIPAN, ACC, UVM, LUAD, THYM, LIHC, TGCT, SARC, SKCM, BRCA. Interestingly, overexpression of RelB could be a protective factor in three cancer types (SARC, SKCM, BRCA) (Figure 3A). The Kaplan-Meier curves in TIMER2.0 database showed that abnormally upregulation of RelB was related with a worse overall survival in ACC, LGG, UVM but a better overall survival in BRCA, SKCM and SARC (Figure 3B), which was consistent with previous results. In addition, we observed that RelB was a risky factor in UVM, THYM, GBM, PAAD, LGG, KIRP, and ACC but a protective factor in SARC and ESCA for PFI (Supplementary Figure S1). As for DFI, RelB was a risky factor in ACC but a good indictor in ESCA (Supplementary Figure S1). To disease free survival (DSS), RelB was a risky factor in UVM, THYM, LUAD, LGG, KIRP, KIRC, GBM, ACC but a protective factor in SKCM and SARC (Supplementary Figure S1). Taken together, RelB was of great value in predicting tumor prognosis.

## 4 DNA methylation and genetic alteration analysis of RelB in pan-cancer

**FIGURE 4:**
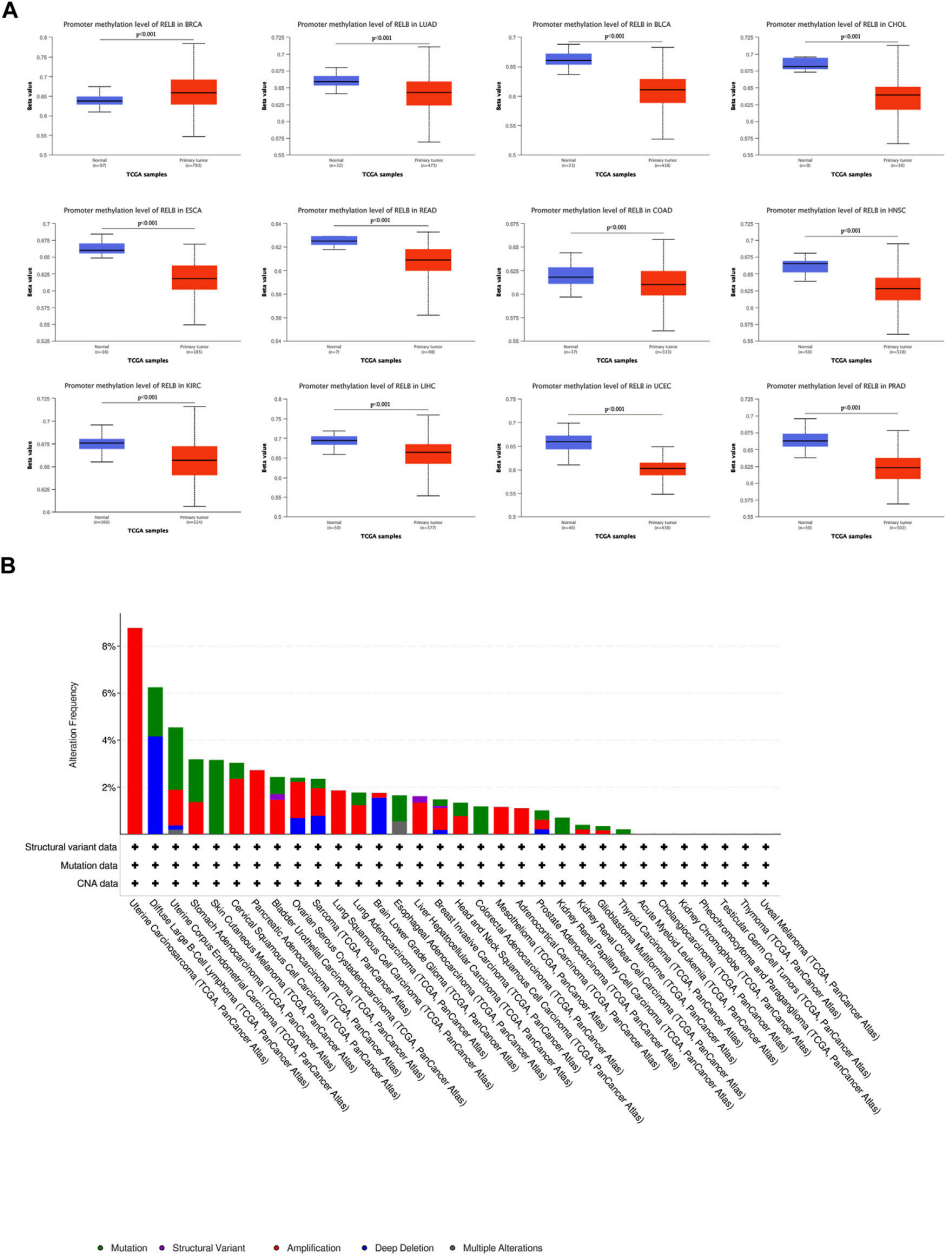
The DNA methylation and mutation profile of RelB in pan-cancer. The Beta value indicates level of DNA methylation ranging from 0 (unmethylated) to 1 (fully methylated). Different beta value cut-off has been considered to indicate hyper-methylation [Beta value: 0.5–0.7] or hypo-methylation [Beta value:0.25–0.3]. **(A)** The promotor methylation level of RelB in human pan-cancer was investigated based on the UALCAN database. **(B)** The mutation profile of RelB was explored and amplification was the major type of mutation.

It was well-known that DNA methylation functioned as an epigenetic modulating mechanism for gene expression (Mahmoud and Ali, 2019). DNA hypermethylation resulted in the loss-of-function for plenty of tumor suppressor genes. DNA methylation is a chemical modification that defines cell type and lineage through the control of gene expression and genome stability and cancer cells are characterized by aberrant DNA methylation and genome-wide hypomethylation. Aberrant DNA methylation is key epigenetic mechanisms associated with tumor initiation, cancer progression, and metastasis (Park and Han, 2019). We therefore investigated the correlation between RelB expression and the level of DNA methylation. According to the TCGA database, DNA methylation level was markedly decreased in LUAD, BLCA, CHOL, ESCA, COAD, HNSC, KIRC, LIHC, LUSC, PRAD, READ and UCEC (Figure 4A) but increased in BRCA (Figure 4A). It was commonly accepted that inactivation of specific tumor-suppressor genes led to a consequence of hypermethylation within the promoter regions and a large number of studies have demonstrated a list of genes silenced by DNA methylation in various cancer types (Kulis and Esteller, 2010). Moreover, we employed cBioPortal database to investigate the genetic alteration of RelB in different cancers (Figure 4B). We observed amplification was the major mutational type of RelB in 32 cancer types based on the results. The highest incidence rate of genetic variation of RelB was found in OV, and amplification was the major type of mutations in most cancers (Figure 4B). Based on the above results, we found the level of DNA methylation was lower, which supported the hypothesis that RelB played an oncogenic role in tumor and was partly valuable in predicting tumor prognosis.

5. RelB-Related Signaling Pathways in Human Pan-Cancer Identified By GSEA

**FIGURE 5:**
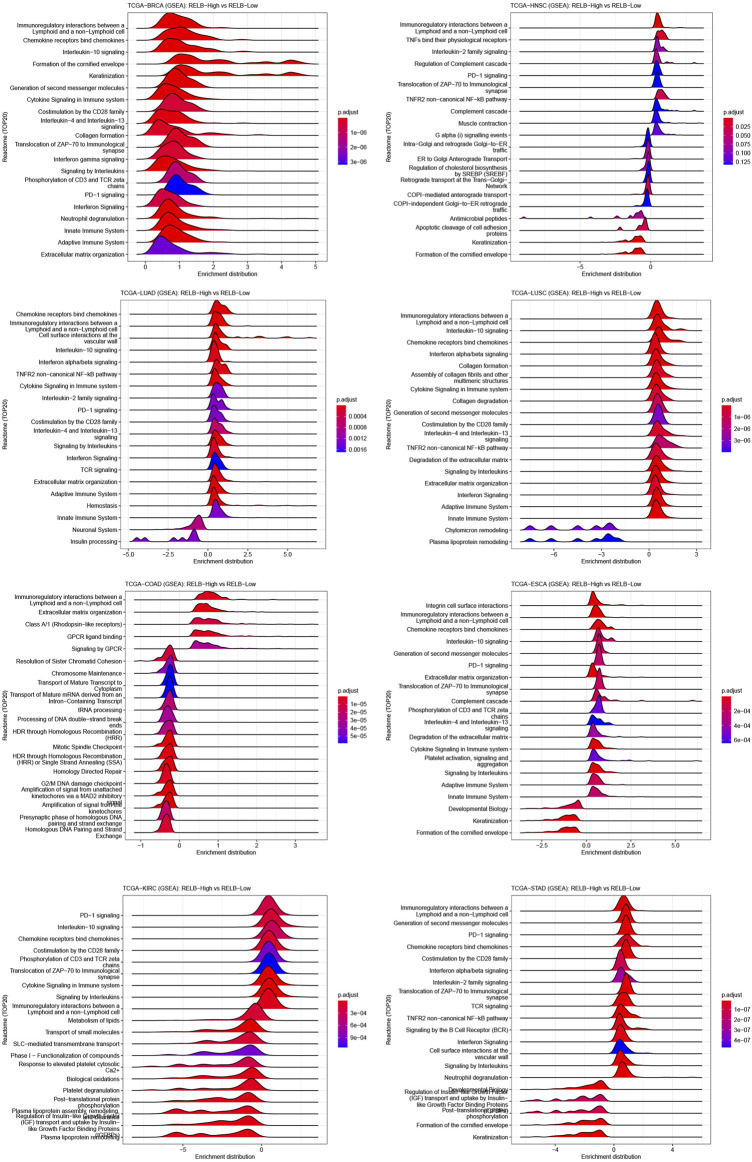
GSEA plot of RelB in human pan-cancer. The top 20 pathways correlated with RelB were shown in the GESA plot in different cancers using the CAMOIP website. RelB was strongly correlated with immunoregulatory interaction between lymphoid and a non-lymphoid cell in most cancer types.

GSEA analyses were performed to ascertain the signaling pathways and molecular mechanism affected by RelB in human cancers. We observed the pathway of immunoregulatory interactions between a lymphoid and a non-lymphoid cell was dramatically activated in most human cancers (Figure 5). Most importantly, increased RelB expression level led to markedly activation of immunity-related pathways, including PD1 signaling, cytokine signaling, TCR signaling and innate and adaptive immune system. Previous studies had demonstrated that RelB was particularly essential for lymphoid organogenesis, B-cell maturation, inflammation and was engaged in modulating tumor immunization (Sun, 2017; Harrington and Annunziata, 2019). Put together, we strongly believed there was a close correlation between RelB expression and tumor immunity based on our findings.

6. Association of RelB Expression and Immune Cell Infiltration in Pan-Cancer

**FIGURE 6:**
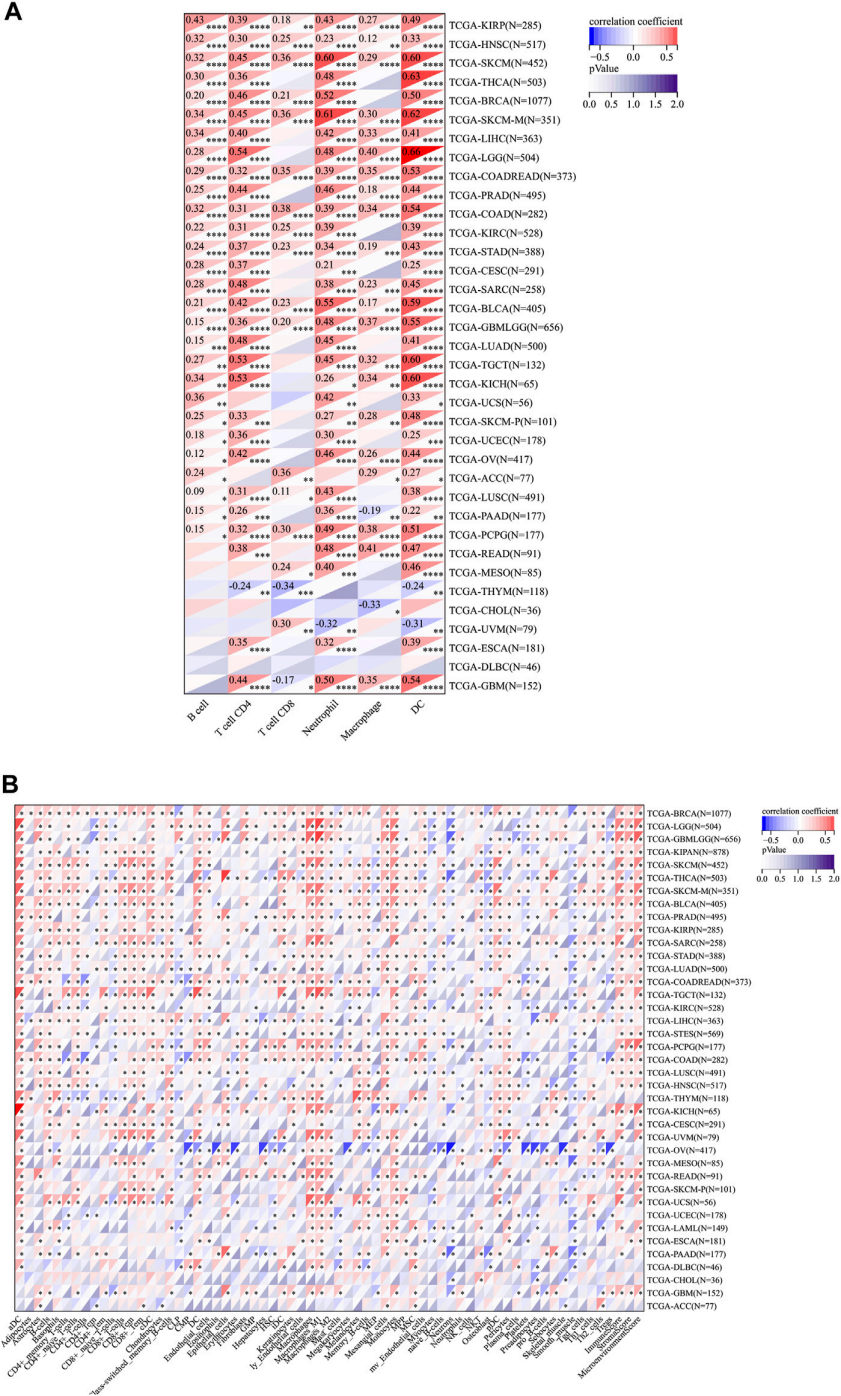
Relationship between RelB expression and the immune cell infiltration level in human pan-cancer; **(A)** Heatmap of correlations of the expression of RelB and the immune infiltration level in all types of human cancer using TIMER database. **(B)** Heatmap of correlations between the expression of RelB and the infiltrated cells in human cancers using xCell. *p < 0.05; **p < 0.01; ***p < 0.001.

These results inspired us that RelB expression might exert profound effects on tumor microenvironment to influence the immunotherapy response. Thus, we paid our attention to the immune cell infiltration across cancers. Different immune-infiltration cells led to various treatment outcomes and overall survival. We then estimated the association between RelB expression and the major types of immune-infiltration cells using the data from Timer2.0 database. The results implied that RelB expression was markedly correlated with the infiltration level of B cells in 28 types of cancer, CD4 T cells in 30 types of cancer, CD8 T cells in 18 types of cancer, neutrophils in 32 types of cancer, macrophages in 23 types of cancer and dendritic cells in 34 types of cancers (Figure 6A). These immune cells are very important for cancer immunotherapy, especially T cells. RelB had a close relationship with them, which implied RelB was associated with cancer immunity. In addition, RelB was positively associated with these six types of immune cells in KIRP, HNSC, SKCM, COAD, STAD, BLCA, PCPG (Figure 6A). To further clarify the relationship between RelB expression and infiltration of 38 subtypes of immune cells, we exploited the xCell algorithm. The results showed RelB expression was negatively correlated with infiltration of most immune cell types in OV (Figure 6B). The tumor immune microenvironment was a complicated ecosystem and the immune-infiltrated cells had a great impact on the prognosis. Based on GSEA results**,** RelB was closely correlated with CD4 T cells, CD8 T cells, B cells, cancer-associated macrophages and DCs, which were important immune-modulating factors for cancer immunity.

7. Relationships Between RelB Expression and Immune Checkpoint Genes, Chemokines, Immunostimulators, and MHC-Related Genes in Pan-Cancer

Immune checkpoints played a vital role in tumor immunotherapy and affected ICB treatment outcome to a great extent. We therefore investigated the correlation between RelB expression and immune checkpoint genes, immunoinhibitors, and immunostimulators (Supplementary Figure S3). We observed that RelB expression was significantly related to most immune checkpoint genes, like PD1, PD-L1, CTLA4, TNFRSF, TGFBR1 and IL10RB in most cancer types (Supplementary Figure S3). Moreover, RelB expression was positively correlated with the expression of chemokines in PAAD, THCA, BRCA, PRAD, KIPAN but negatively associated with the expression of chemokines in BLCA, COAD, READ. Besides, RelB expression was also positively correlated with the expression of immunostimulators in LUAD, KIPAN, STAD, STES and other cancer types. RelB expression was positively correlated with most MHC-related genes in almost all cancer types. In summary, RelB expression was closely correlated with tumor immune-modulating genes.

8. Relationships Between RelB Expression and TMB and MSI in Pan-Cancer

**FIGURE 7:**
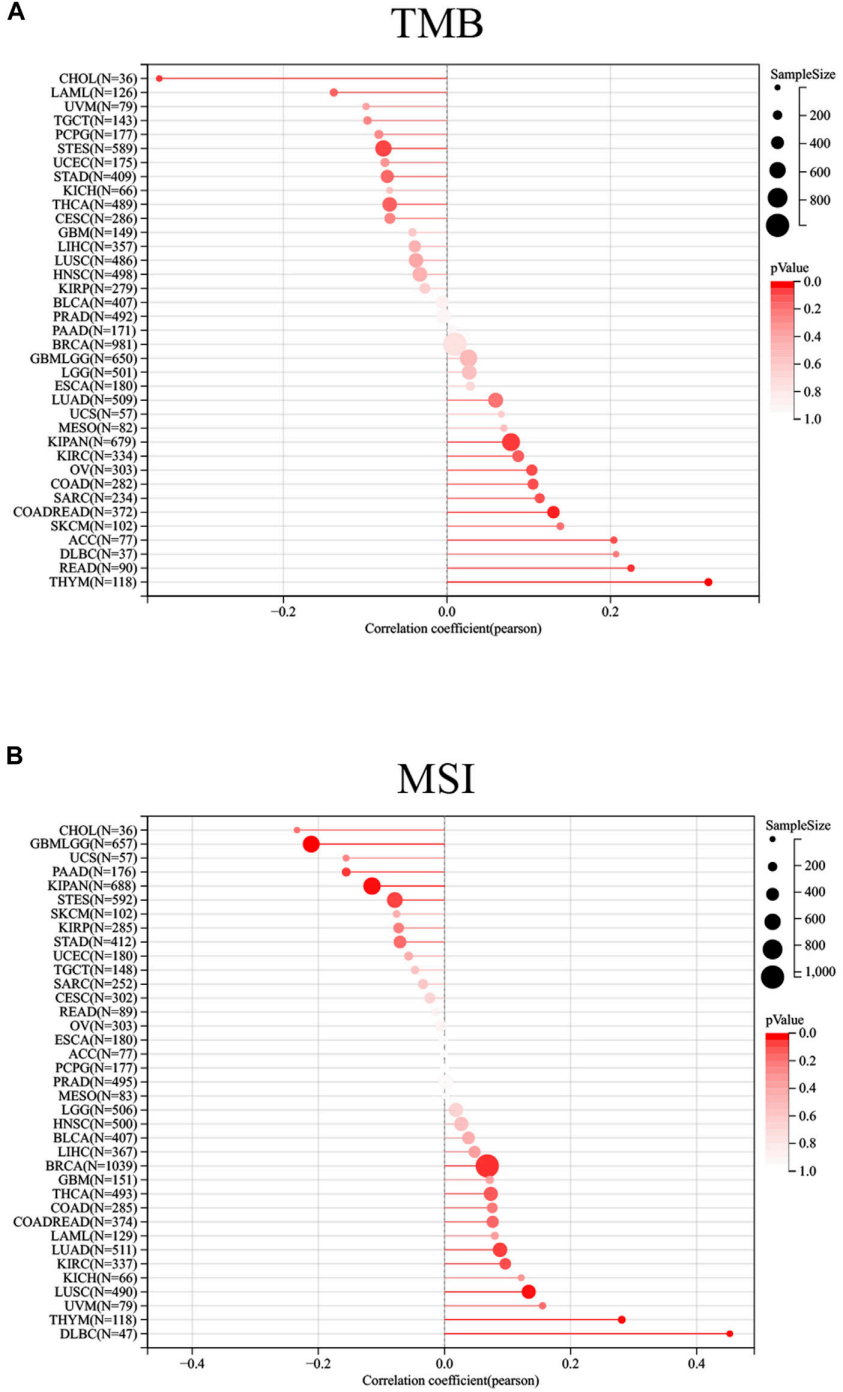
Relationship between RelB expression and TMB and MSI; Correlation of RelB expression with TMB, MSI in human pan-cancer was shown in the lollipop graph **(A)** and **(B)**.

A growing body of evidence suggested that TMB and MSI could function as two effective biomarkers in predicting immunotherapy responses (Rizzo et al., 2021). Therefore, we investigated the relationship between RelB expression and the level of TMB/MSI. We obtained the data from UCSC website (https://xenabrowser.net/), and we then calculated their correlation using Pearson method. The result suggested that RelB expression was positively correlated with the level of TMB in KIPAN, THYM, READ, COADREAD but negatively associated with the level of TMB in CHOL (Figure 7A). In addition, the correlation of RelB expression with MSI was checked and explored. The result showed that RelB expression had a significantly positive correlation with the level of MSI in LUAD, BRCA, LUSC, THYM, DLBC but had a negatively correlation with the level of MSI in KIPAN, PAAD, GBMLGG (Figure 7B).

9. Effect of Heat RelB Expression on the Expression of Immune Checkpoints

**FIGURE 8:**
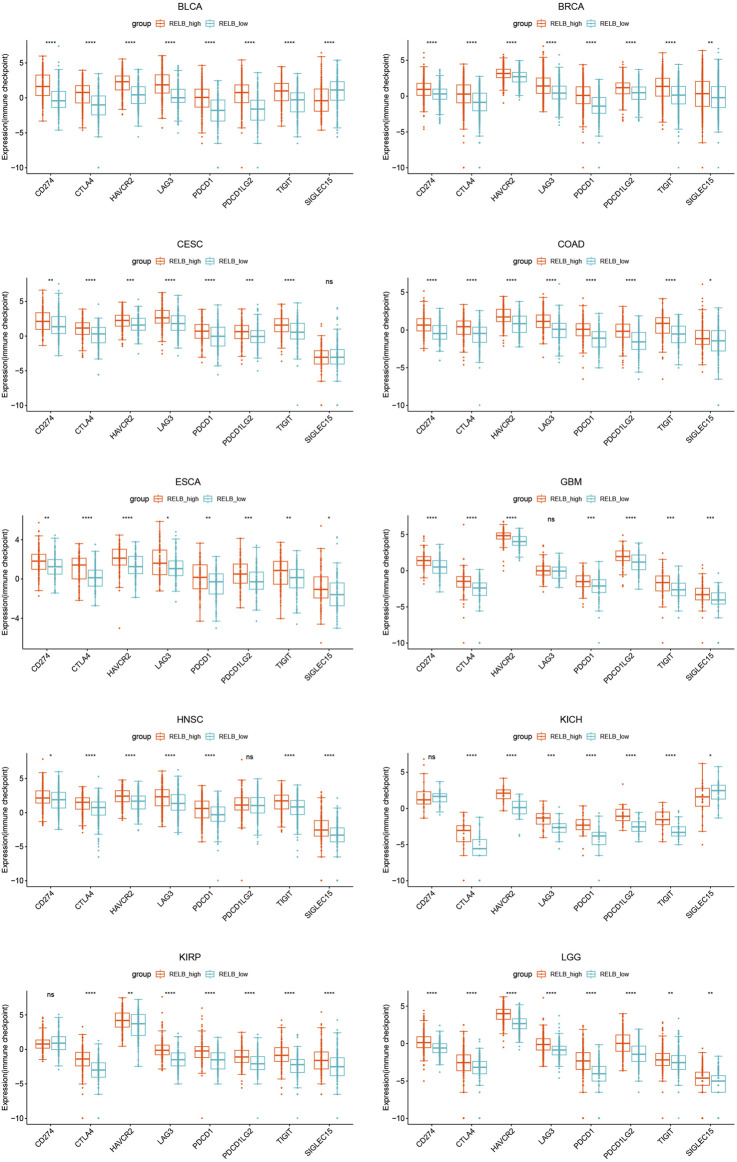
The expression of classical immune checkpoints between the RelB low-expression group and the high-expression group in human pan-cancer. These immune checkpoints were significantly upregulated in all human cancers. *p < 0.05, **p < 0.01, ***p < 0.001, ****p < 0.0001.

The patients were divided into two groups based on the median value of RelB expression (RelB^high^ and RelB^low^ group). The effect of RelB expression on the typical immune checkpoints including CD274, CTLA4, HAVCR2, LAG3, PDCD1, PDCD1LG2, TIGHT and SIGLEC15 was evaluated. These genes were consistently upregulated in the RelB high-expression group in all cancer types (Figure 8; Supplementary Figure S4), which suggested RelB was strongly correlated with immune blockade therapy.

10. RelB Was a Promising Drug-Target in Human Pan-Cancer

**FIGURE 9:**
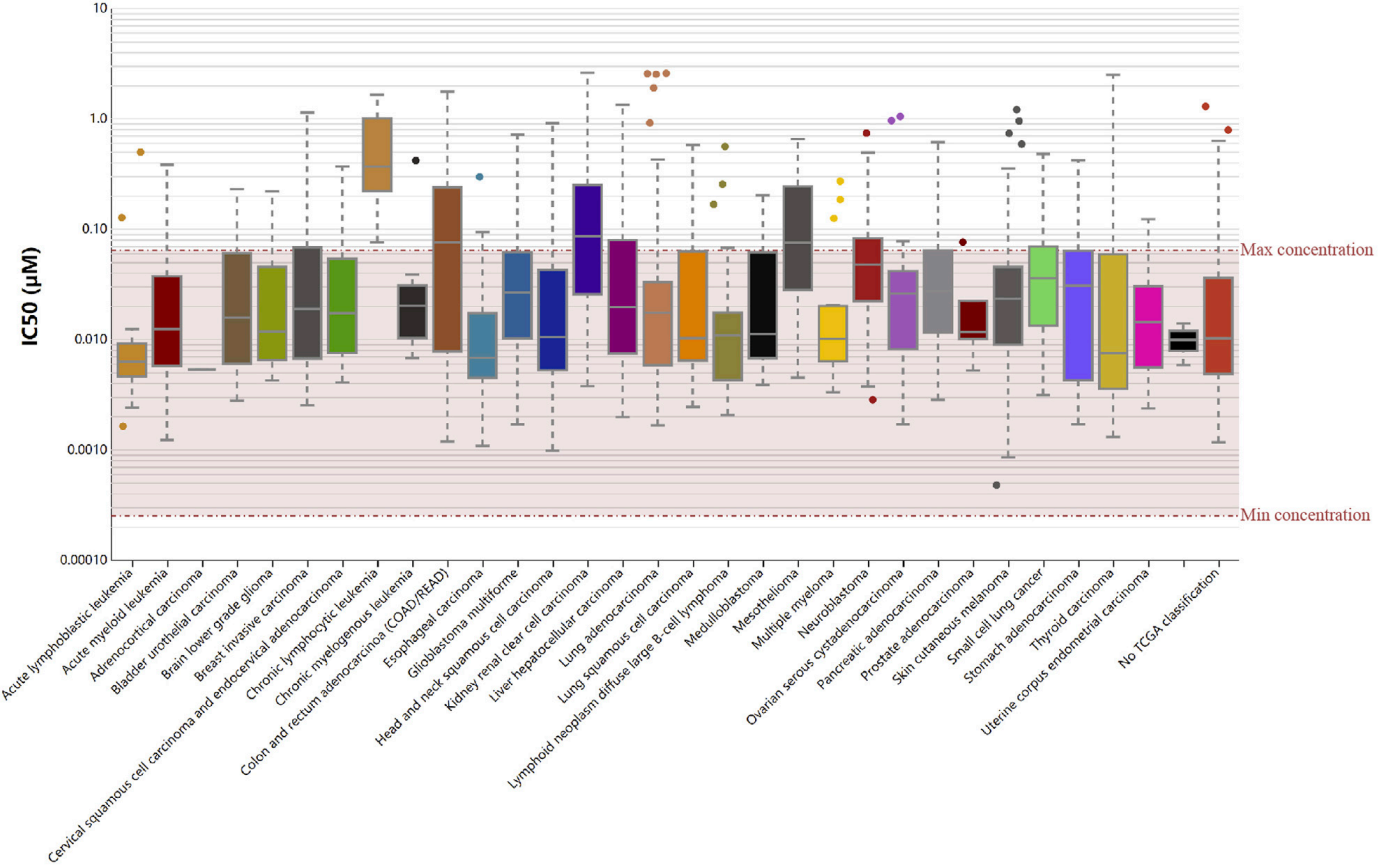
RelB was a sensitive drug-target in human pan-cancer using the Genomic Drug of Sensitive and IC50 of Vinorelbine was checked in human pan-cancer.

We then evaluated if RelB could serve as an effective drug-target using the Genomics of Drug Sensitivity in Cancer (GDSC) database. Based on the results from GDSC website, Vinorelbine was an effective inhibitor for RelB. Vinorelbine was a microtubule destabiliser and affected the mitosis of cancer cells in human cancers. The IC50 was checked in human pan-cancer (Figure 9) and we found the majority of human patients in human pan-cancer were sensitive to Vinorelbine. Taken together, RelB could be a drug target to affect tumor immunotherapy.

## 5 Discussion

Cancer has gradually been a serious threat to public health recent years in the worldwide due to its high morbidity and mortality. Early detection and diagnosis are beneficial for improving the treatment outcome. There are many available cancer treatments, including surgical resection, radiation and chemotherapy, but their efficacy is still limited. It is very urgent to identify novel biomarkers to precisely predict the response of cancer treatments. Therefore, we analyzed the pan-cancer data downloaded from TCGA database to identify the immune-related biomarkers and its molecular mechanisms involved in tumor initiation and progression.

RelB, a member of NF-kappaB family, took participate in many biological processes including inflammation, immune responses, tumorigenesis and lymphocyte development. However, its exact role in pan-cancer tumor immunization is unclear and needs to be clarified. In this study, we applied a list of bioinformatics methods to explore the potential tumor-promoting or tumor-suppressing role of RelB via investigating the significant correlation between RelB expression and the overall survival (OS), DNA methylation, TMB, MSI, MSS, immune-infiltration cells and classical immune checkpoint genes in human pan-cancer.

Our study was the first systematic analysis of RelB to verify its role in immune responses in pan-cancer. The results showed that RelB was significantly upregulated from three aspects of gene, mRNA and protein respectively in various cancers, which was consistent with previous clinical trials and some basic studies (Chaiswing et al., 2022; Li et al., 2022). These results showed that RelB was engaged in oncogenesis in pan-cancer. The alternative NF-kappaB subunit RelB dampened the effects of ionizing radiation by stimulating expression of the mitochondria-localized antioxidant enzyme manganese superoxide dismutase in prostate cancer (Holley et al., 2010). RelB also contributed to cancer immune evasion by inhibiting T cell immunity via the amplification of PD1/PD-L1 mediated immune checkpoint in prostate cancer (Zhang et al., 2022). Besides, RelB was reported to confer DLBCL cell resistance to DNA damage-induced apoptosis in response to doxorubicin and RelB positivity was correlated with high expression of cellular inhibitor of apoptosis protein (clAP2) (Eluard et al., 2022). In addition, RelB signaling pathway promoted senescence-like growth arrest in breast cancer (Costa et al., 2019). Previous study suggested RelB served as an oncogenic role and delivered chemo-resistance to 5-FU, and RelB was thought to be a potential prognostic maker in CRC (Zhou et al., 2018). In summary, RelB served as an oncogene in the process of tumor genesis in different cancers.

In order to check if RelB could be a reliable prognostic biomarker for human cancers, we analyzed the RelB expression in different clinical stages in pan-cancer. The distribution of RelB expression was various but commonly higher in almost all pathological stages. Besides, we investigated the correlation of RelB expression with the relevant prognostic indictors including OS, PFI, DFI, DSS in pan-cancer. We observed that higher expression of RelB was a risky threat in most cancer types but a protective factor in SARC, SKCM, BRCA. Taken together, RelB was a promising prognostic marker for predicting cancer risk.

DNA methylation was reported to be a Universe mechanism to control the gene expression. We thus evaluated the relationship between RelB expression and promotor methylation level. The results showed the promotor methylation level was significantly lower in cancer tissues, which implied RelB was an oncogenic role in pan-cancer. To illustrate the molecular mechanism by which RelB influenced pan-cancer, GSEA analyses were performed. These results implied that RelB greatly affected these pathways involved in oncogenesis. Otherwise, RelB also impacted the immunoregulatory interaction between lymphoid cell and a non-lymphoid, which attracted our attention to the relationship between RelB expression and TIME. It was noteworthy that RelB could involve in many biological processes, including PD1 signaling, cytokine signaling, TCR signaling and innate and adaptive immune system **based on the results of GSEA analysis**. Meanwhile, RelB correlated with the tumor genesis related pathway, suggesting RelB promote the process of tumor genesis in human pan-cancer.

A growing body of evidence showed tumor immune microenvironment played an assignable role in the clinical outcomes and response to the therapy. Tumor-infiltration immune cells could be exploited by cancer cells to profoundly enhance or dampen the effectiveness of cancer therapy to influence tumor progression by taking pro- and anti-tumorigenic actions (Chen and Mellman, 2017). We then focused on the relationship between RelB expression and tumor-infiltrating immune cell or tumor immune microenvironment. Previous study showed tumor dampened the immune response and immunotherapy by making use of immune checkpoint genes, including PD1, PD-L1 and CTLA4 (Kruger et al., 2019). Recently, cancer immunotherapy, aimed to enhance antitumor immune responses, had changed the paradigm for cancer treatment (Rosenberg, 2014). However, challenges followed because only a small proportion of cancer patients showed good response to immunotherapy. Based on the fact that the efficacy of immunotherapy was limited, it was desperately urgent to identify novel targets or biomarkers to improve the efficacy of immunotherapy. RelB is an effective transcription factor with pleiotropic functions in immune responses. The activation of RelB was previously associated with the activation and function of T cells. Besides, RelB was greatly engaged in Tconv cell homeostasis, activation and proliferation, and for their polarization toward different favors of Thelper cells *in vitro* (Lalle et al., 2021). T cells were the key point for cancer immunotherapy. RelB-activated alternative NF-κB signaling has been shown to promote antigen presentation by DCs, which further results in excessive immune cell infiltration (Sun, 2017). Tumor-infiltration immune cells consist of T cells, B cells, dendritic cells, macrophages, cancer-associated fibroblasts and neutrophiles. To further explore the relationship between RelB and TIME, we evaluated the correlation of RelB expression and the abundance of these different immune infiltration cells. RelB expression was significantly linked with the relative abundance of CD4 T cells, CD8 T cells, B cells, neutrophils, macrophages and dendritic cells in most cancer types. We then used xCEll algorithm to ascertain the relationship between RelB and the immune-infiltration cells. We observed RelB expression was positively correlated with the abundance of aDC, macrophages and monocytes in pan-cancer. TAMs played a vital role in the tumor-related processes, including metastasis, angiogenesis, and immunosuppression in different cancers (Quail and Joyce, 2013; Ngambenjawong et al., 2017; Anderson et al., 2021). TAMs were generally divided into two functional contrasting groups, namely, classical activated M1 macrophages and alternatively activated M2 macrophages (Pan et al., 2020). M1 macrophages exhibited antitumor functions, including mediating cytotoxicity and antibody-dependent cell-mediated cytotoxicity to effectively kill cancer cells. On the contrary, M2 macrophages promoted the occurrence and metastasis of cancer cells. A better understanding of their polarization from an antitumor phenotype to a pro-tumor phenotype was needed to prompt us reevaluate its clinical significance as a biomarker in human cancer (Duan and Luo, 2021). Here, the results showed RelB was positively correlated with the abundance of the infiltrating macrophages and monocytes, which displayed the complexity of the tumor immune environment. Moreover, increasing evidence suggested dendritic cells represented only a small proportion of the tumor microenvironment cells but functioned as an antitumor weapon (Sadeghzadeh et al., 2020; Gerhard et al., 2021). Our results also demonstrated RelB expression was significantly related to most immunoinhibitors, different chemokines, immunostimulators and MHC-associated genes.

We observed RelB expression was linked with TMB and MSI in some cancer types. Cancers with high level of TMB were more possible to generate immunogenic peptides, therefore altering the efficacy of immunotherapy. High frequencies of MSI were thought to result in genomic instability, which was a stimulator for the initiation of cancers. We found MSS-related genes were strongly expressed in OV and ACC. Previous study also implied MSI was a predictor of sensitivity in immunotherapy-based cases (Baretti and Le, 2018). In this study, they were used to predict the immunotherapy response. Consequently, RelB was a potential target for immunotherapeutic strategy. Because RelB was a biomarker for cancer prognosis, we then investigated if there were any drugs that particularly targeted RelB in GDSC database. The result showed Vinorelbine was an effective inhibitor for RelB, and most cancers were sensitive to it.

However, the limitation was still in existence based on these bioinformatic analyses. First, some contradictory results of individual cancers were uncovered. Second, the sample size was small and we needed to test and verify the expression and function of RelB in a larger sample cohort. In the end, *in vitro* or *in vivo* experiments were needed to verify these conclusions and immune cell infiltration in cancer patients requires strong evidences in the clinic trials. In one word, RelB was abnormally expressed in different cancer types and was closely associated with tumor immunity. According to our analyses, we believed RelB was a potential molecular biomarker in cancer immunotherapy in most cancer types including ACC, BRCA, ESCA, KIPAN, COAD, PRAD, STAD, HNSC, LUSC, LUAD, LIHC, BLCA, THCA, OV, PAAD and CHOL.

## Figures and Tables

**Table:** 

**ACC**	Adrenocortical Carcinoma
**BLCA**	Bladder Urothelial Carcinoma
**BRCA**	Breast Invasive Carcinoma
**CESC**	Cervical Squamous cell Carcinoma and Endocervical adenocarcinoma
**CHOL**	Cholangiocarcinoma
**COAD**	Colon adenocarcinoma
**READ**	Rectum adenocarcinoma Esophageal carcinoma
**DLBC**	Lymphoid Neoplasm Diffuse Large B-cell Lymphoma
**ESCA**	Esophageal carcinoma
**GBM**	Glioblastoma multiform
**GBMLGG**	Glioma
**HNSC**	Head and neck squamous cell carcinoma
**KICH**	Kidney chromophobe
**KIPAN**	Pan-kidney cohort (KICH + KIRC + KIRP)
**KIRC**	Kidney renal clear cell carcinoma
**KIRP**	Kidney renal papillary cell carcinoma
**LAML**	Acute myeloid leukemia
**LGG**	Brain lower grade glioma
**LIHC**	Liver hepatocellular carcinoma
**LUAD**	Lung adenocarcinoma
**LUSC**	Lung squamous cell carcinoma
**MESO**	Mesothelioma
**OV**	Ovarian serous cystadenocarcinoma
**PAAD**	Pancreatic adenocarcinoma
**PCPG**	Pheochromocytoma and Paraganglioma
**PRAD**	Prostate adenocarcinoma
**READ**	Rectum adenocarcinoma
**SARC**	Sarcoma
**STAD**	Stomach adenocarcinoma
**SKCM**	Skin cutaneous melanoma
**STES**	Stomach and esophageal carcinoma
**TGCT**	Testicular germ cell tumor
**THCA**	Thyroid carcinoma
**THYM**	Thymoma
**UCEC**	Uterine corpus endometrial carcinoma
**UCS**	Uterine carcinosarcoma
**UVM**	Uveal melanoma
**OS**	Osteosarcoma
**ALL**	Acute lymphoblastic leukemia

## Data Availability

Publicly available datasets were analyzed in this study. This data can be found here: https://xenabrowser.net/datapages/?dataset=Tcga Target Gtex_rsem_gene_tpm&host=https%3A%2F%2Ftoil.xenahubs.net &re moveHub=https%3A%2F%2Fxena.treehouse.gi.ucsc.edu%3A443 https ://xenabrowser.net/datapages/?dataset=tcga_RSEM_gene _tpm&host=https%3A%2F%2Ftoil.xenahubs.net&remove Hub=https%3A%2F%2Fxena.treehouse.gi.ucsc.edu%3A443.
