# Supplementary material for: RelB is a potential molecular biomarker for immunotherapy in human pan-cancer
Source: Front Mol Biosci. 2023 Jun 14;10:1178446. doi: 10.3389/fmolb.2023.1178446 (PMC10303125; doi:10.3389/fmolb.2023.1178446)
Supplement: Supplementary file 1 [file Table1.DOCX]

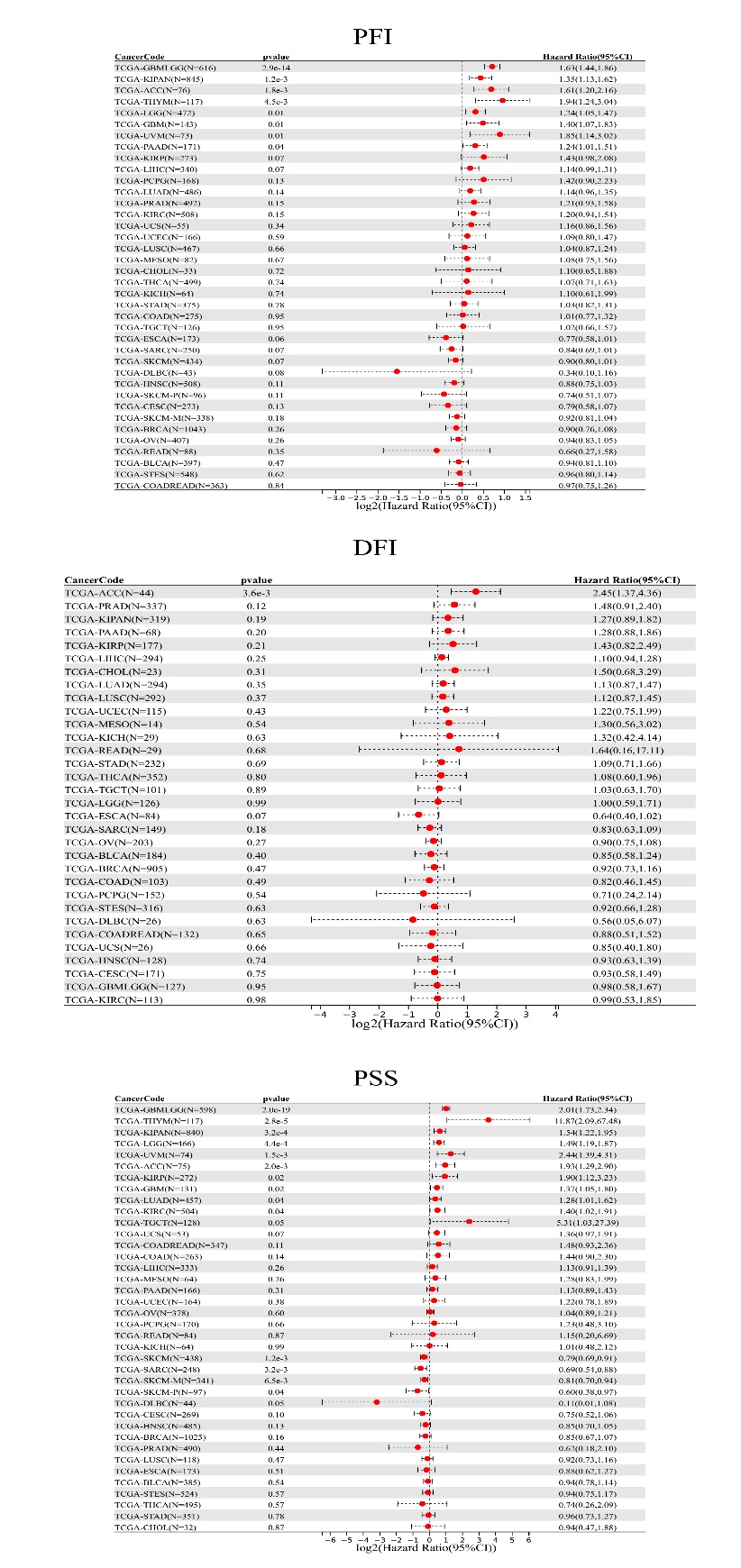


**Supplementary Figure 1** | The correlation of RelB with PFI, DFI, DSS in human pan-cancer was investigated and was shown respectively.


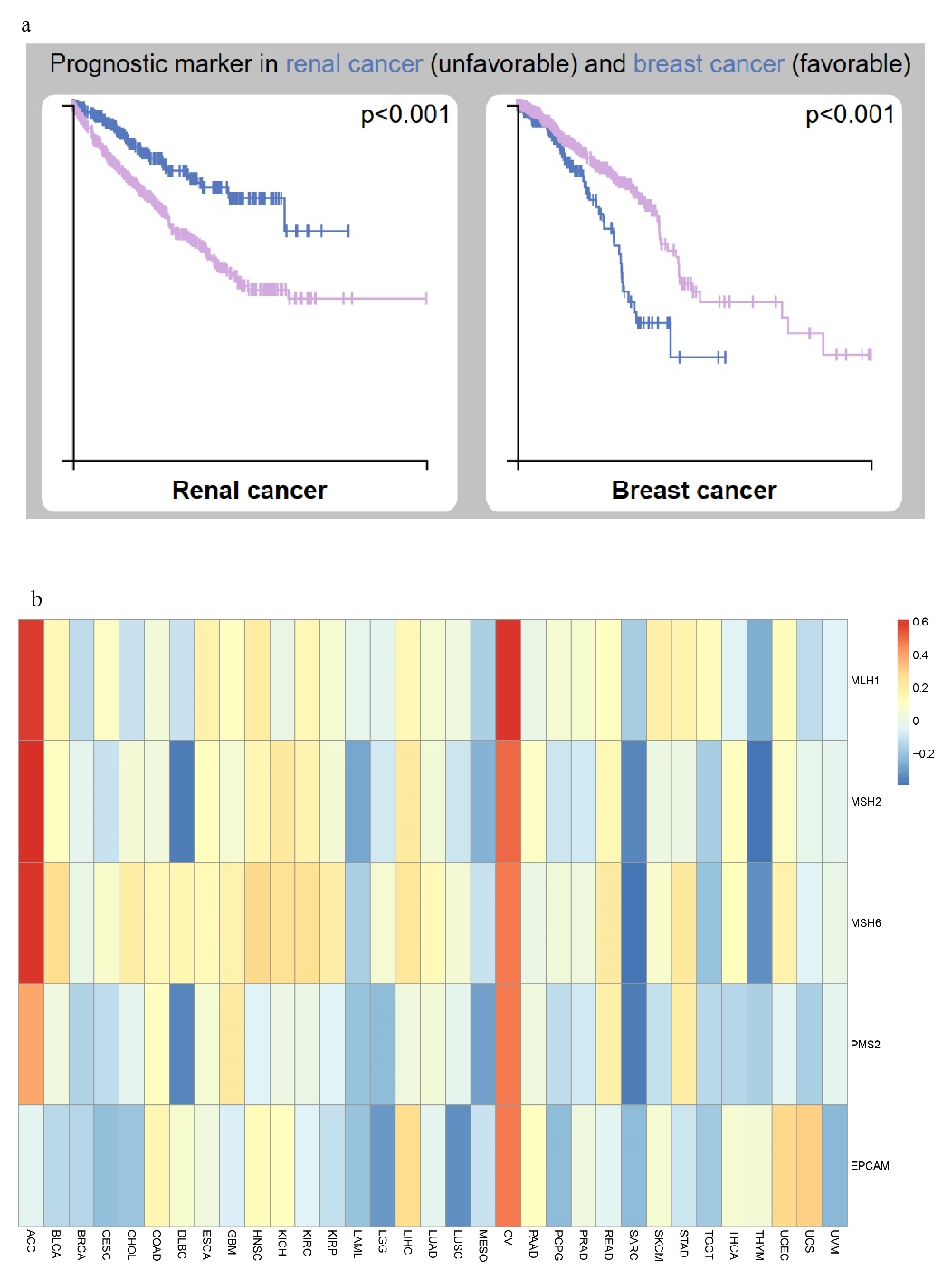


**Supplementary Figure 2** | RelB was a prognostic biomarker in some cancer types. (a)According to the Human Protein Altas database，RelB was an unfavorable prognostic factor in renal cancer but a protective factor in breast cancer; (b) Correlation of RelB expression with the MSS-related genes was evaluated;


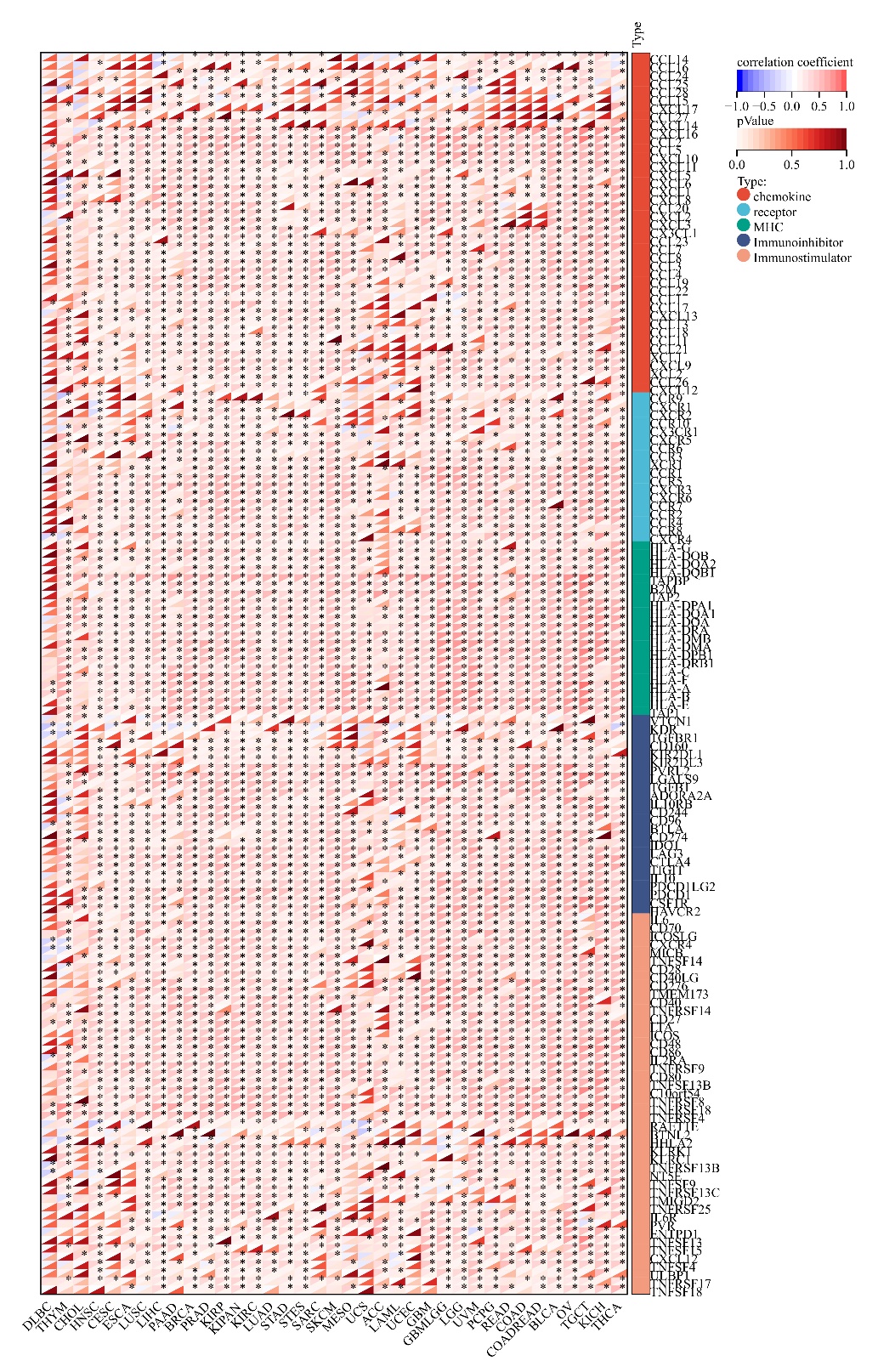


**Supplementary Figure 3|**Correlation between RelB expression and immune checkpoint associated genes, chemokines, immunostimulators, and MHC-related genes

in human pan-cancer.


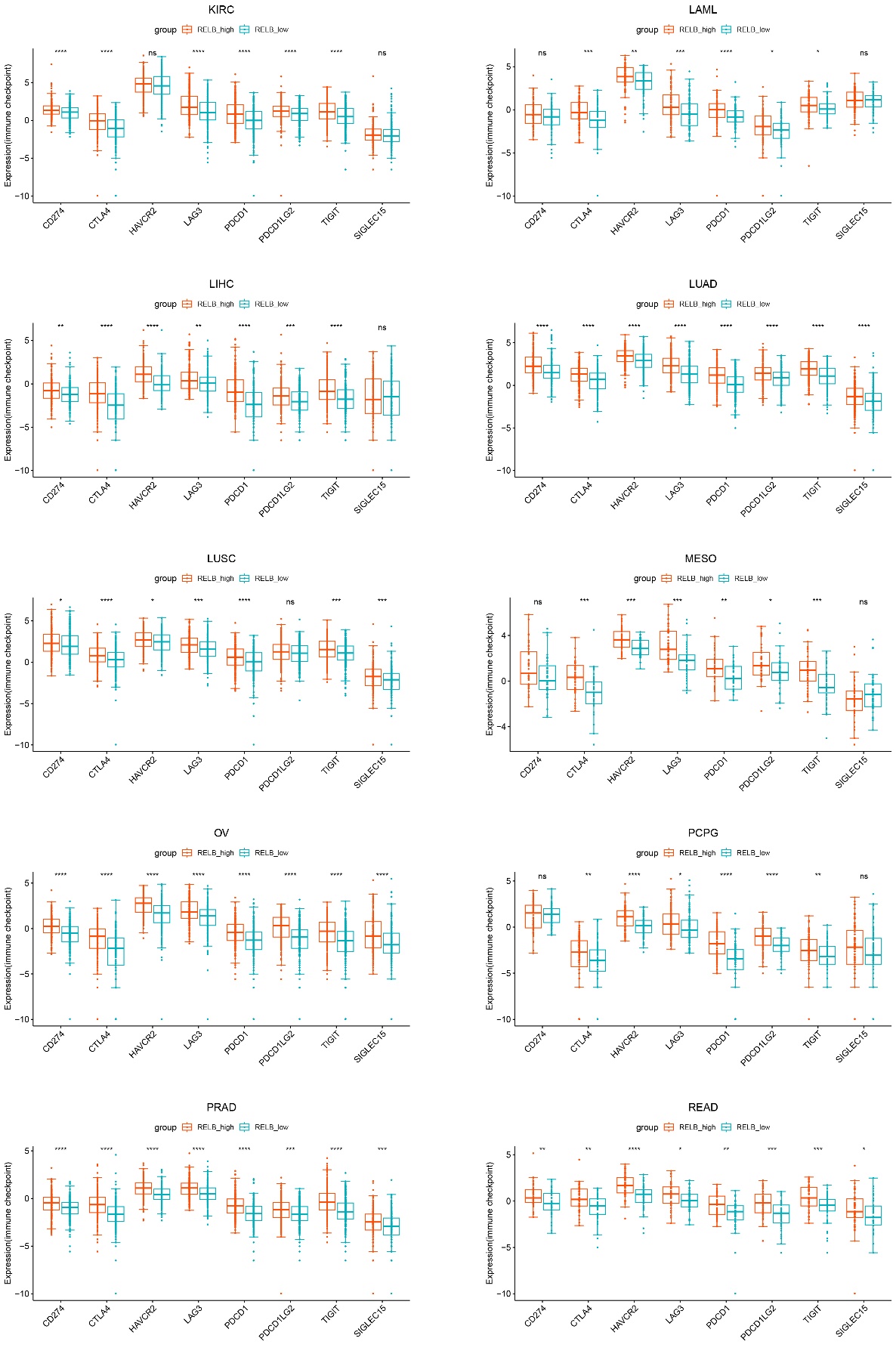


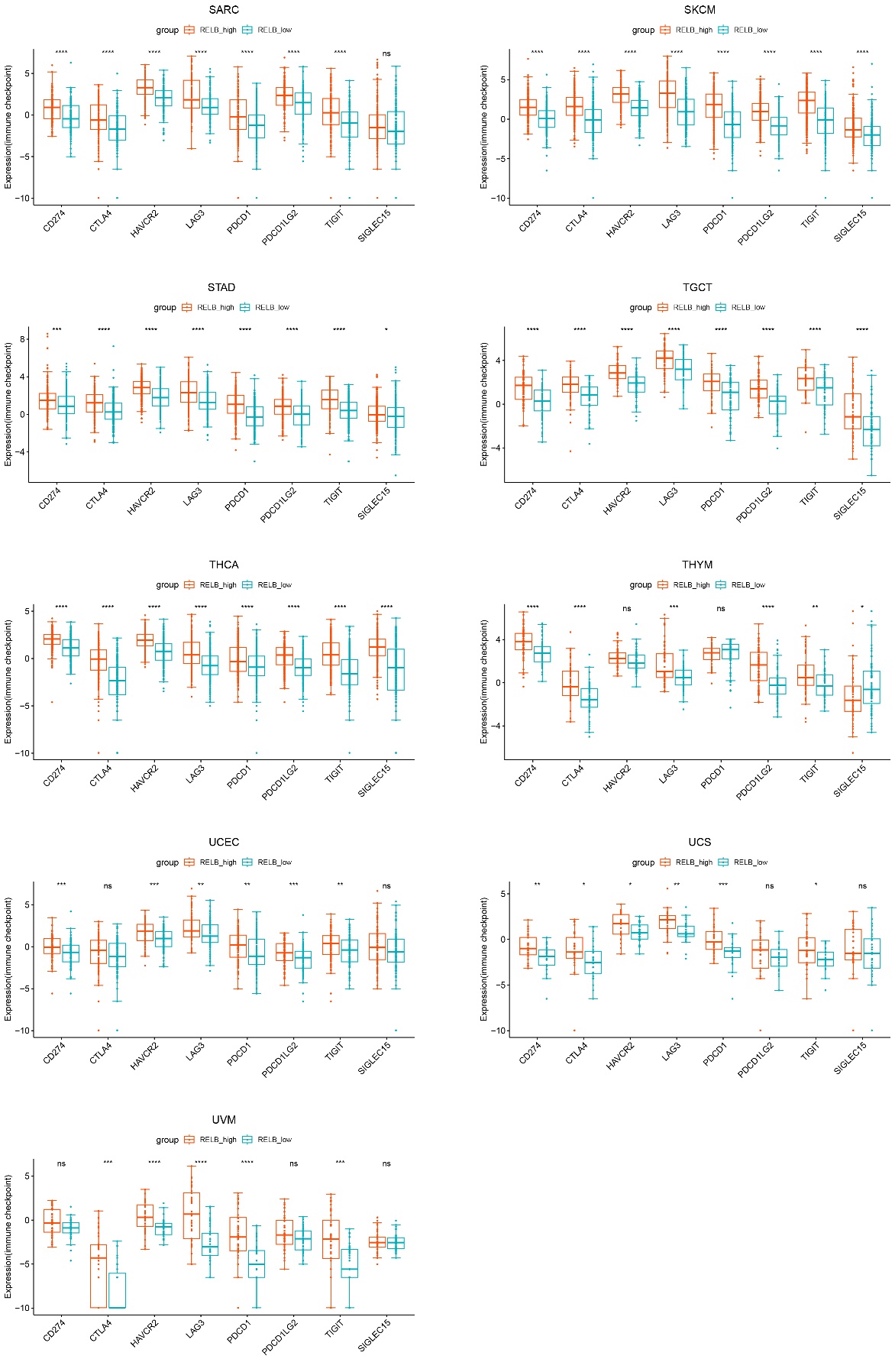


**Supplementary Figure 4|** The expression of classical immune checkpoints between the RelB low-expression group and the high-expression group in human pan-cancer. These immune checkpoints were significantly upregulated in all human pan-cancer. *p < 0.05, **p < 0.01, ***p < 0.001, ****p<0.0001.
